# Supplementary figures and images for: Genomic transfers help to decipher the ancient evolution of filoviruses and interactions with vertebrate hosts
Source: PLoS Pathog. 2024 Sep 3;20(9):e1011864. doi: 10.1371/journal.ppat.1011864 (PMC11398700; doi:10.1371/journal.ppat.1011864)

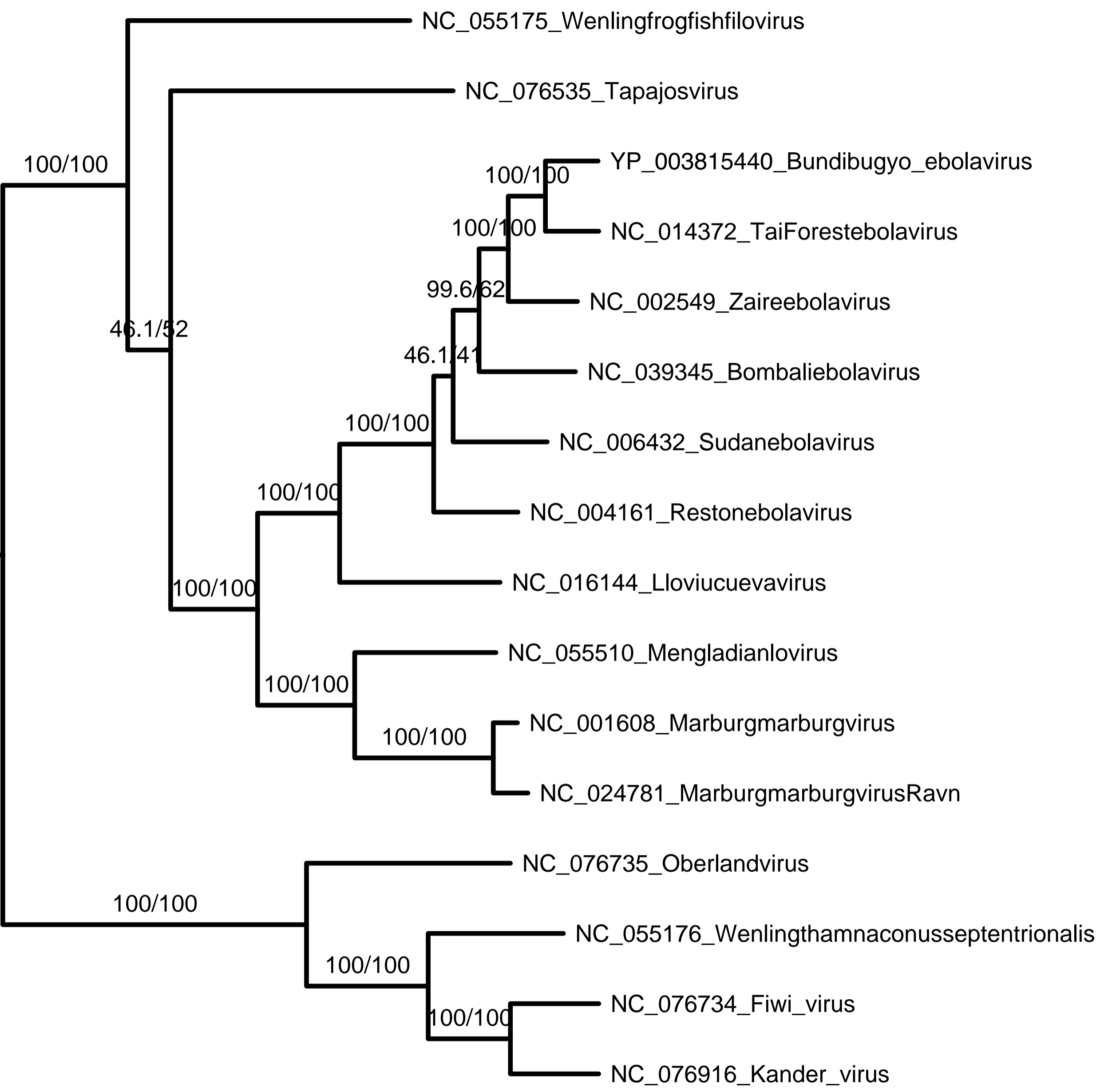

1.0

Supplement: S1 Fig — The substitution model was partitioned by the three codon positions. Genbank accession numbers are part of tip names. Numbers on branches represent approximate likelihood ratio test values and bootstrap values. (PDF) [file ppat.1011864.s001.pdf]

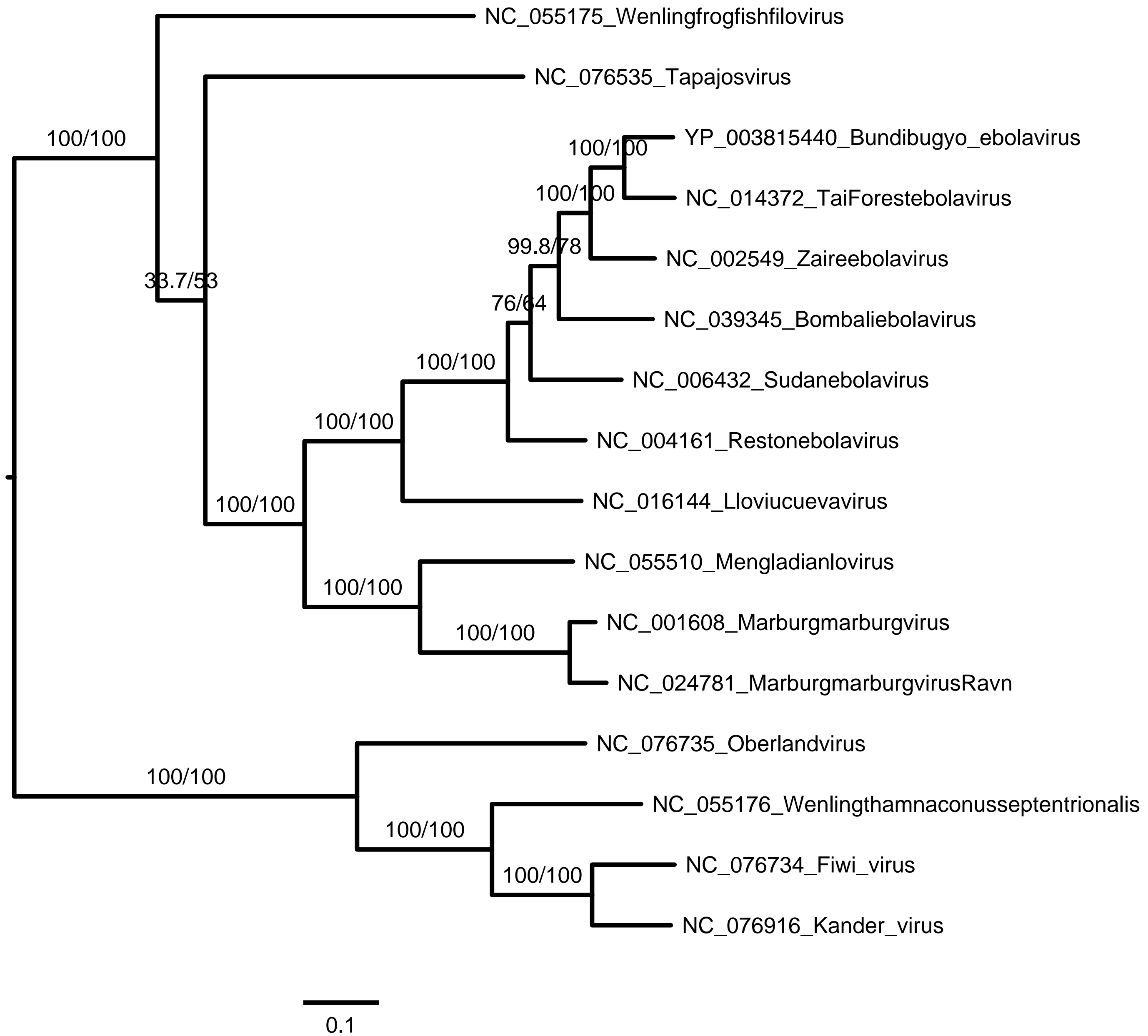

Supplement: S2 Fig — The substitution model was partitioned by the first two codon positions, while the third codon position was omitted. Genbank accession numbers are part of tip names. Numbers on branches approximate likelihood ratio test values and bootstrap values. (PDF) [file ppat.1011864.s002.pdf]

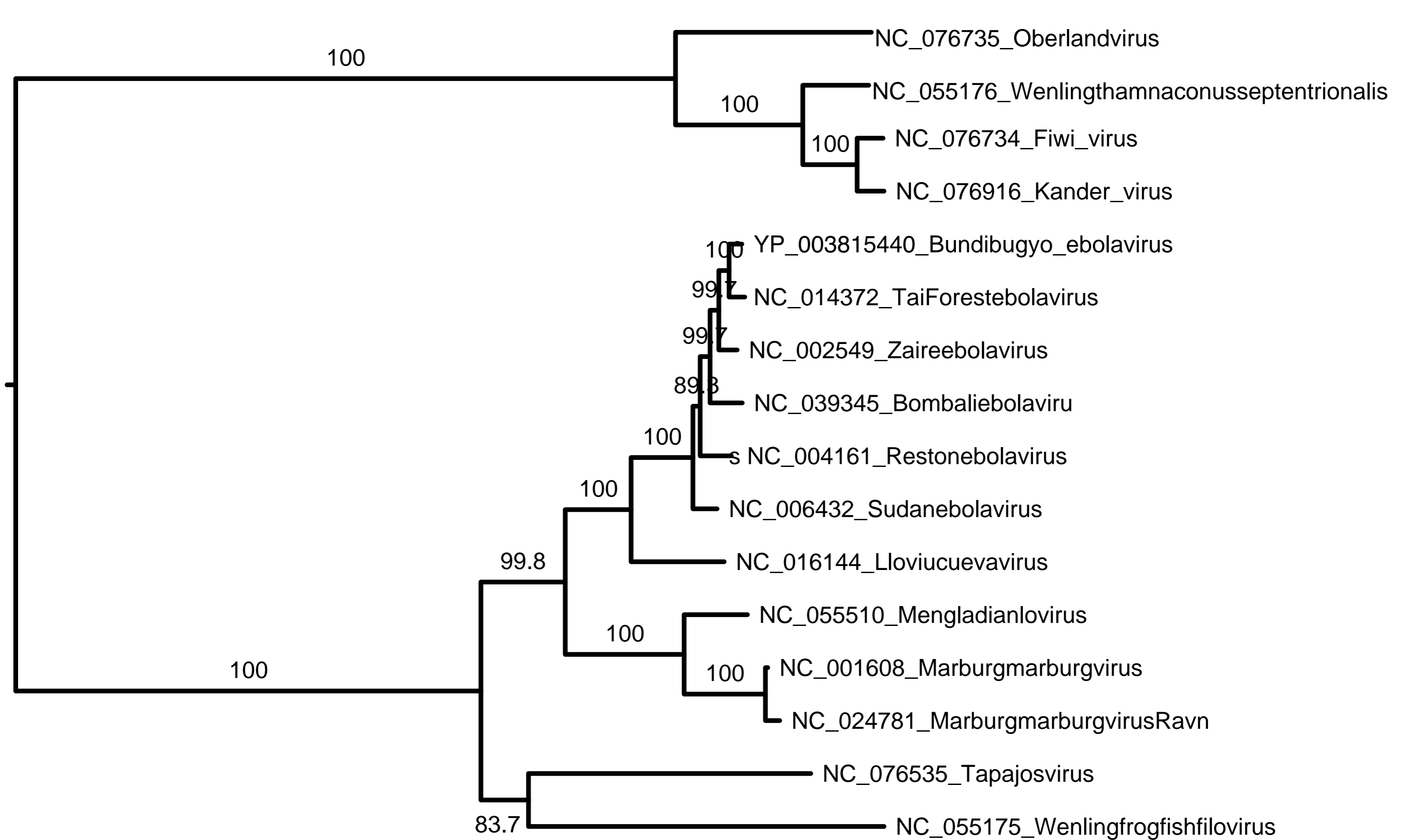

2.0

Supplement: S3 Fig — Genbank accession numbers are part of tip names. Numbers on branches approximate likelihood ratio test values and bootstrap values. (PDF) [file ppat.1011864.s003.pdf]

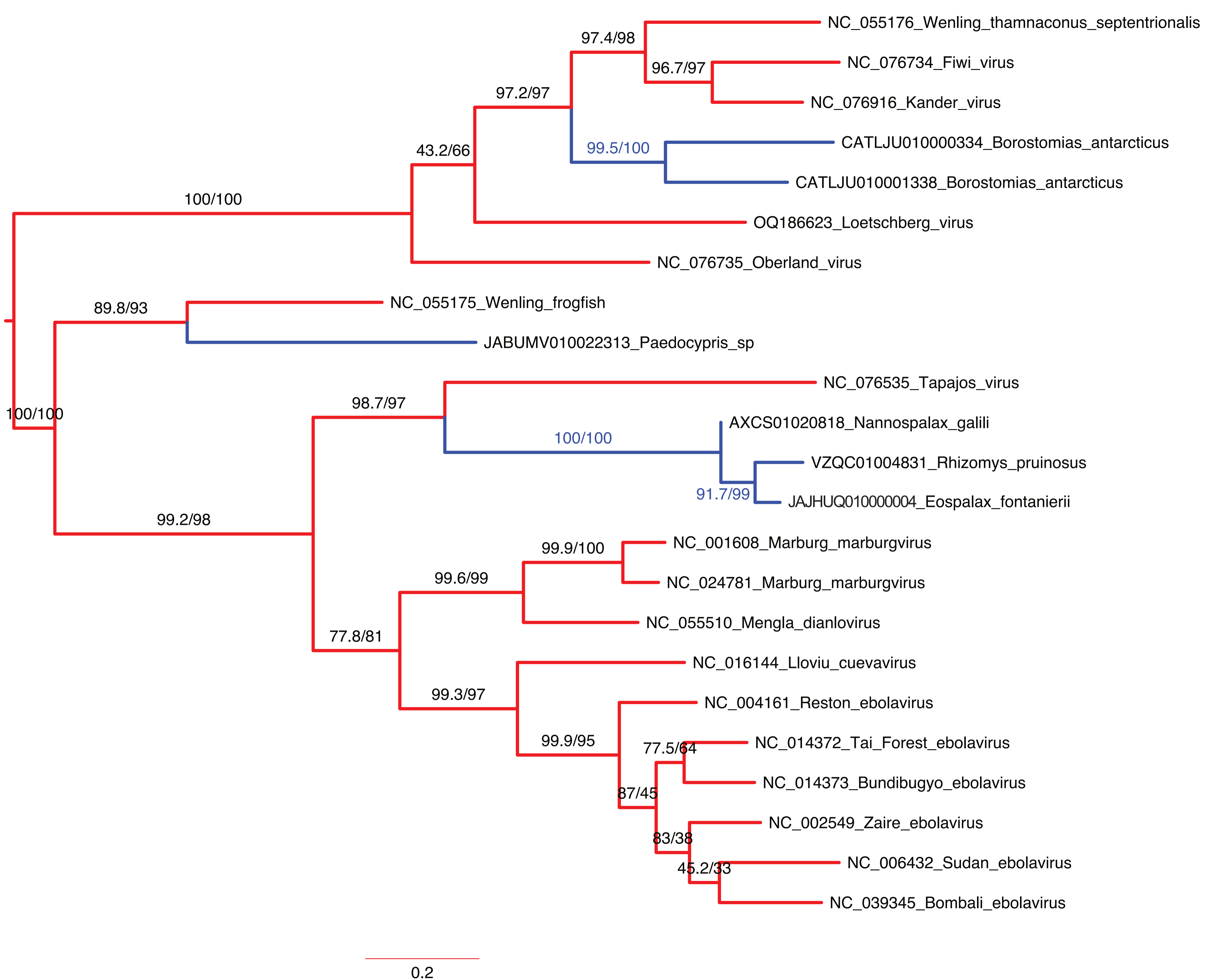

Supplement: S4 Fig — The substitution model was partitioned by the first two codon positions with third codon positions being omitted. Genbank accession numbers are part of tip names. Numbers on branches approximate likelihood ratio test values and bootstrap values. Blue lines indicate branches leading to paleoviruses from vertebrate genomes with extended open reading frames. (PDF) [file ppat.1011864.s004.pdf]

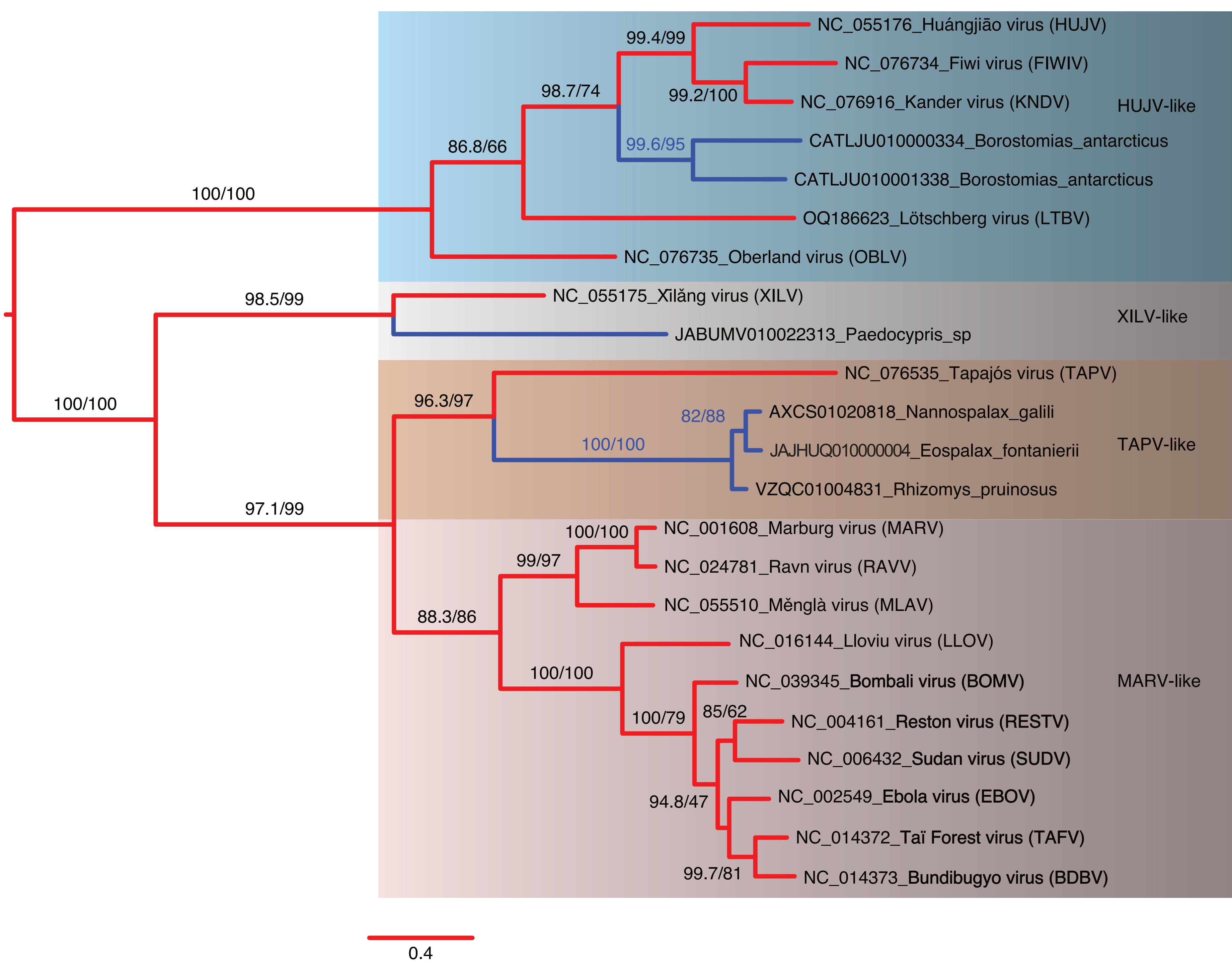

Supplement: S5 Fig — The substitution model was partitioned by three codon positions. Genbank accession numbers are part of tip names. Numbers on branches approximate likelihood ratio test values and bootstrap values. Blue lines indicate branches leading to paleoviruses from vertebrate genomes with extended open reading frames. (PDF) [file ppat.1011864.s005.pdf]

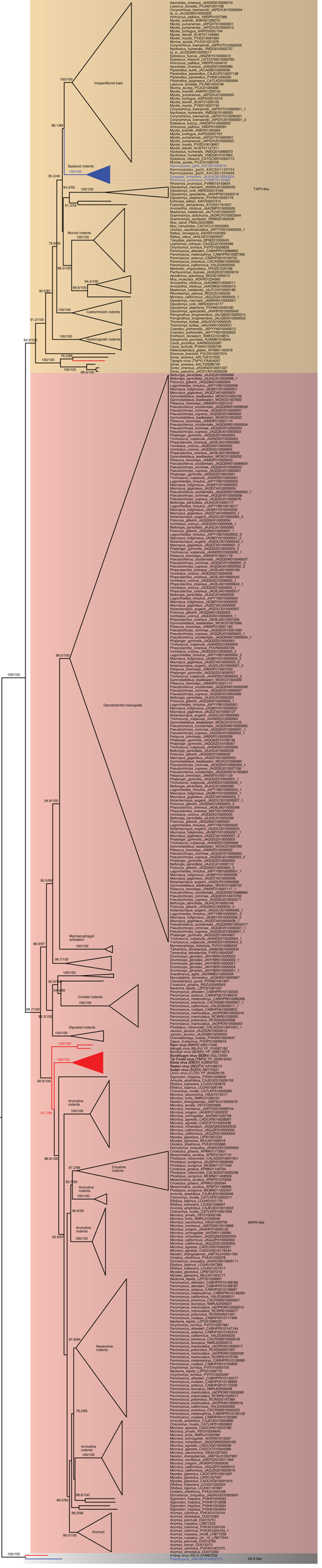

Supplement: S6 Fig — Red lines indicate viral lineages, blue lines indicate vertebrate sequences with open reading frames and black lines indicate vertebrate paleoviral sequences that have disrupted open reading frames. Numbers on branches represent approximate likelihood ratio test values and bootstrap values. Three major clades are shown in shaded rectangles. (PDF) [file ppat.1011864.s006.pdf]

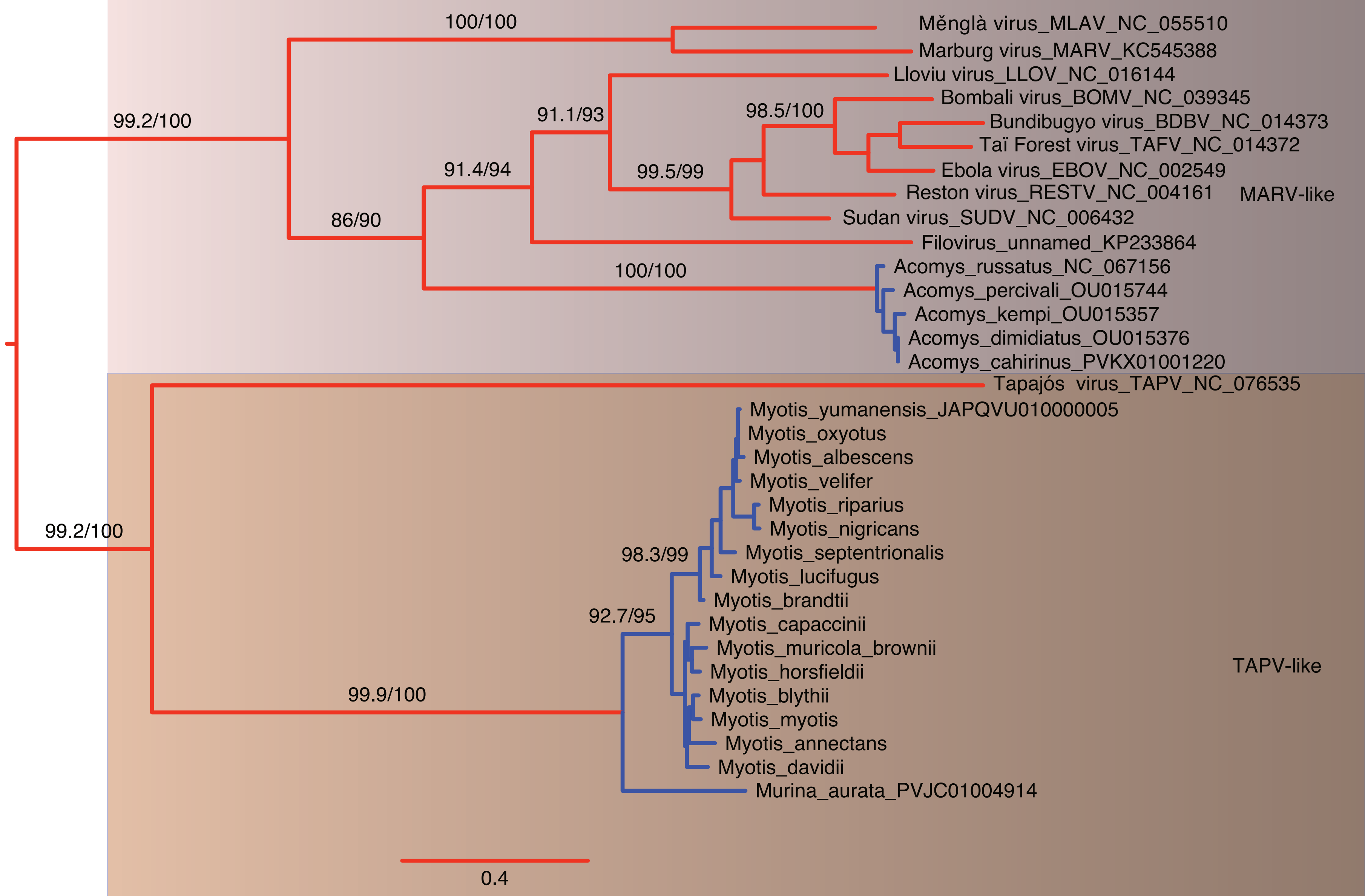

Supplement: S7 Fig — Two major clades (MARV-like and TAPV-like) are identified and shaded. Numbers represent approximate likelihood ratio test values and bootstrap values. Genbank accession numbers are part of tip names. Additional accession numbers for Myotis sp. are MH431024.1-MH431036.1, ALWT01033109.1, and ANKR01158691.1. (PDF) [file ppat.1011864.s007.pdf]

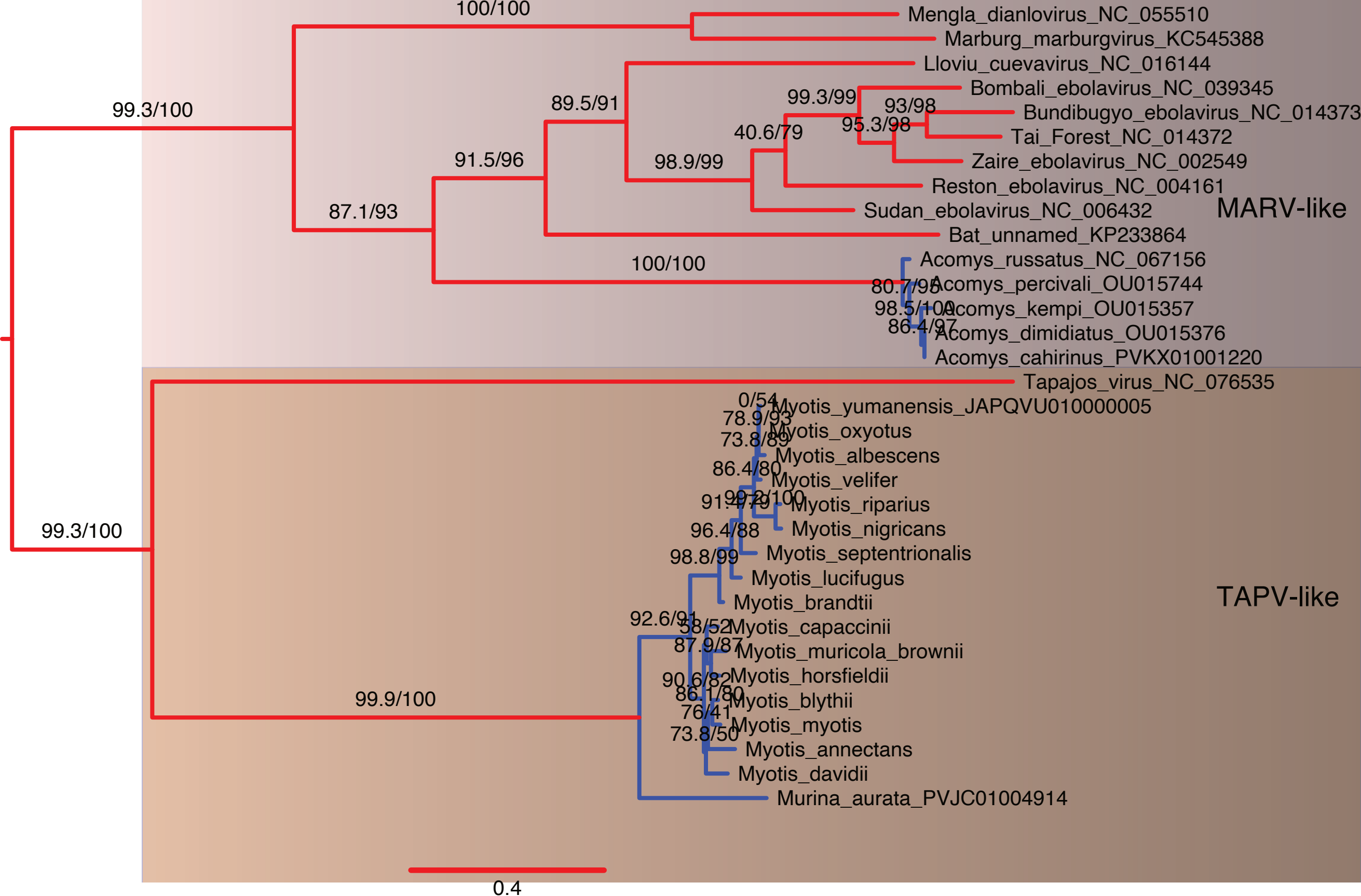

Supplement: S8 Fig — The alignment was filtered using clipKIT. Two major clades (MARV-like and TAPV-like) are identified and shaded. Numbers represent approximate likelihood ratio test values and bootstrap values. Additional accession numbers for Myotis sp. are MH431024.1-MH431036.1, ALWT01033109.1, and ANKR01158691.1. (PDF) [file ppat.1011864.s008.pdf]

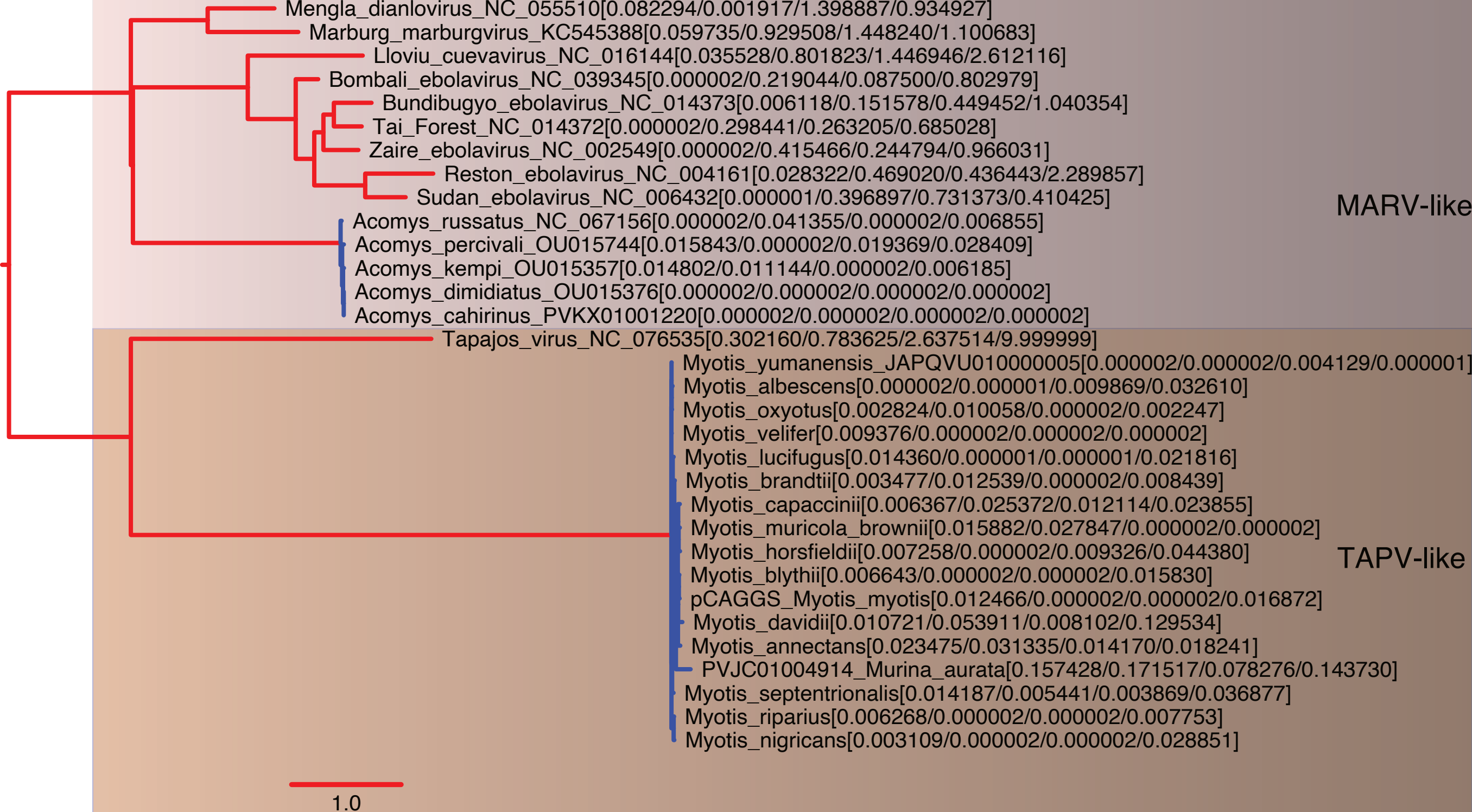

Supplement: S9 Fig — The tree was midpoint rooted and based on a substitution model that specifically accounts for heterotachy (within site rate variation). Additional accession numbers for Myotis sp. are MH431024.1-MH431036.1, ALWT01033109.1, and ANKR01158691.1. (PDF) [file ppat.1011864.s009.pdf]

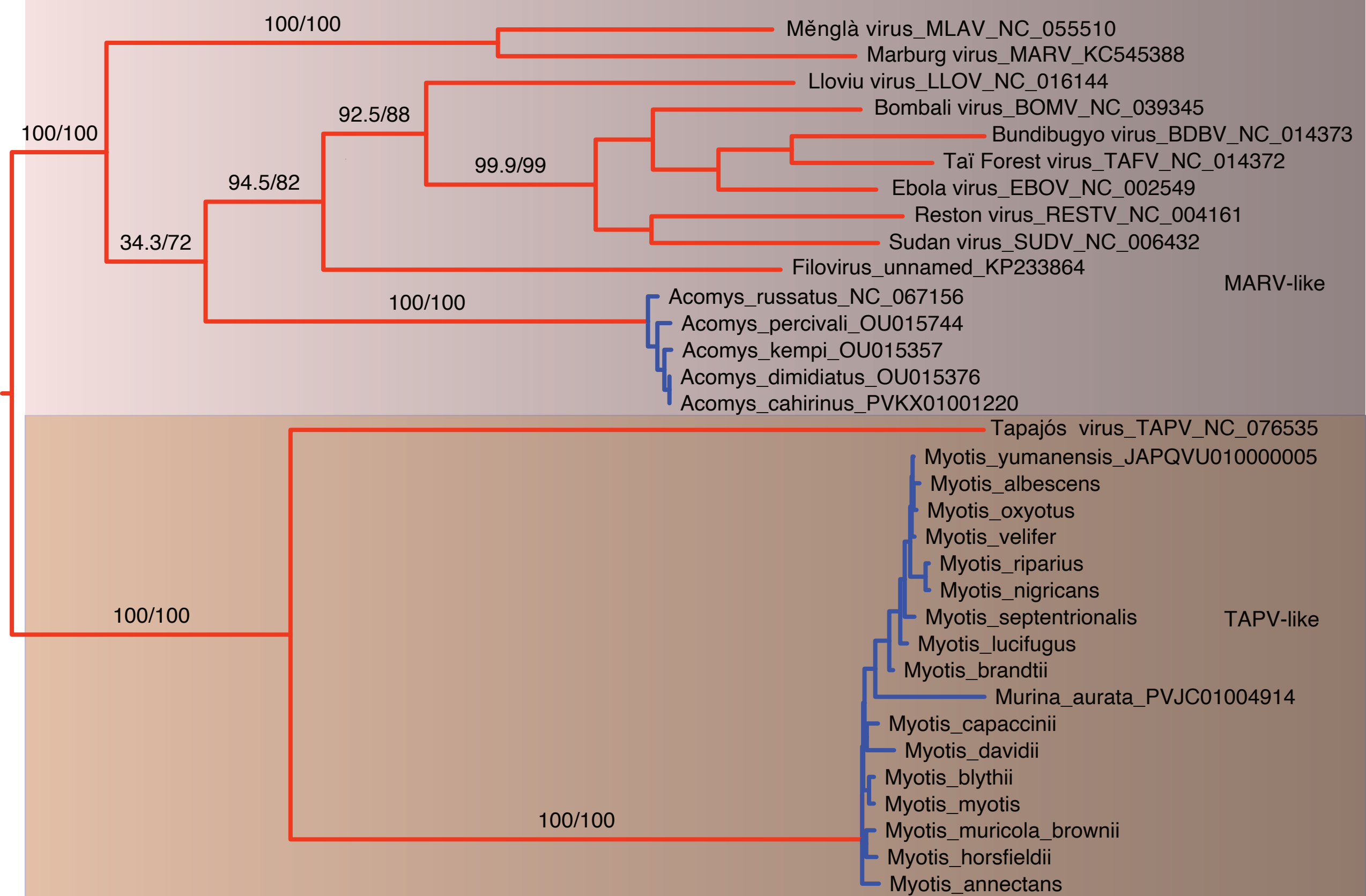

0.3

Supplement: S10 Fig — The substitution model was partitioned by codon position. Two major clades are identified. Numbers represent approximate likelihood ratio test values and bootstrap values. Additional accession numbers for Myotis sp. are MH431024.1-MH431036.1, ALWT01033109.1, and ANKR01158691.1. (PDF) [file ppat.1011864.s010.pdf]

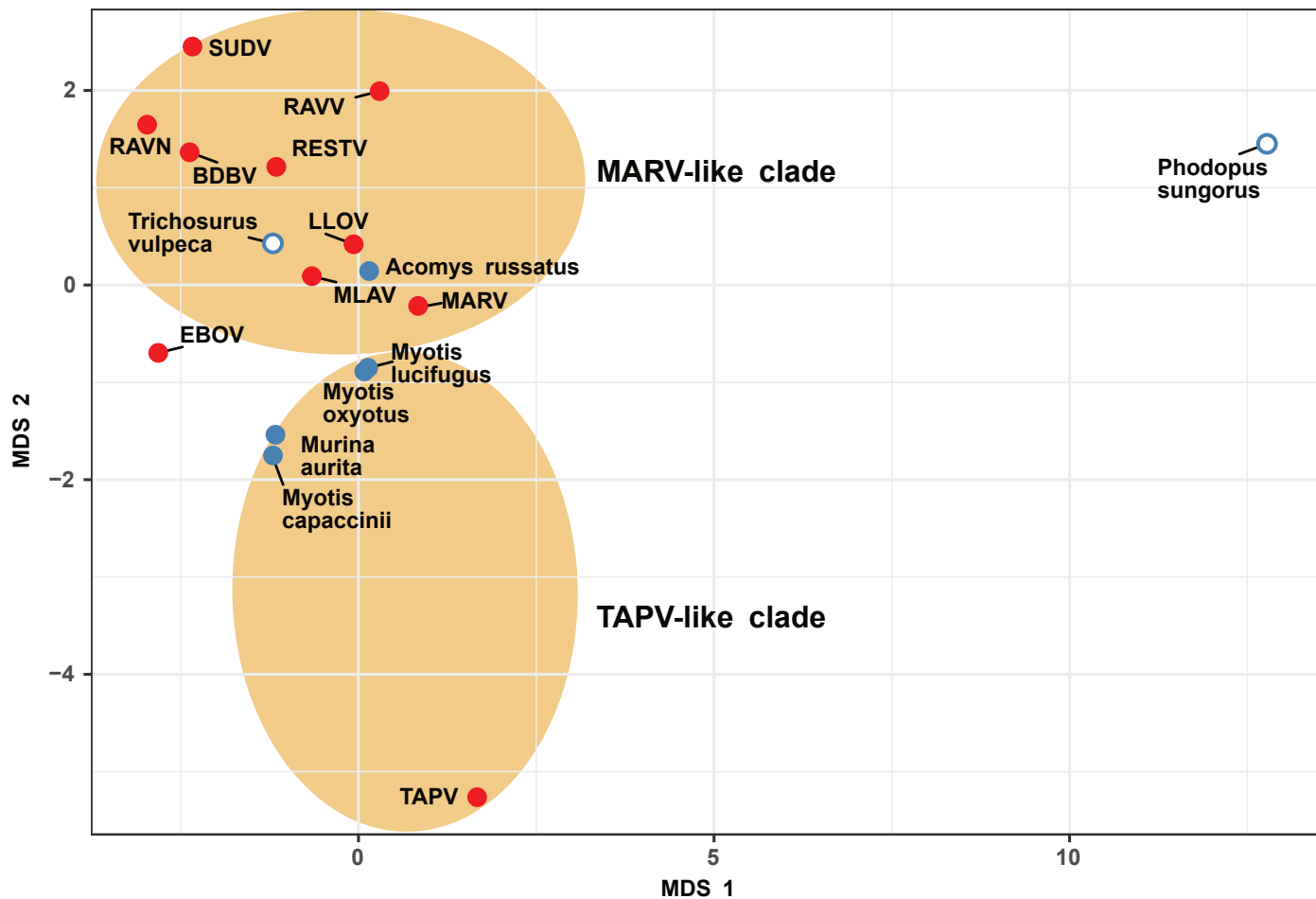

Supplement: S11 Fig — Ovals indicate major clades found in phylogenetic analyses. Red shaded stimuli are based on viral structures while blue stimuli are predicted from vertebrate genome sequences. Solid shading indicates open reading frames are present. (PDF) [file ppat.1011864.s011.pdf]

A.

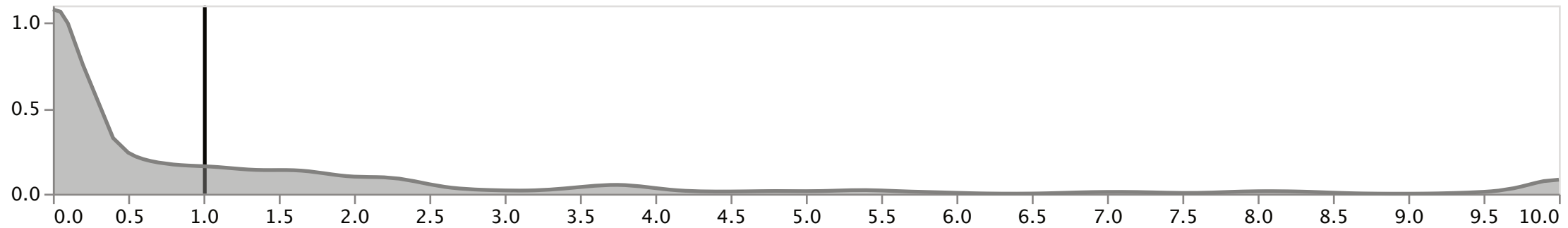

B.

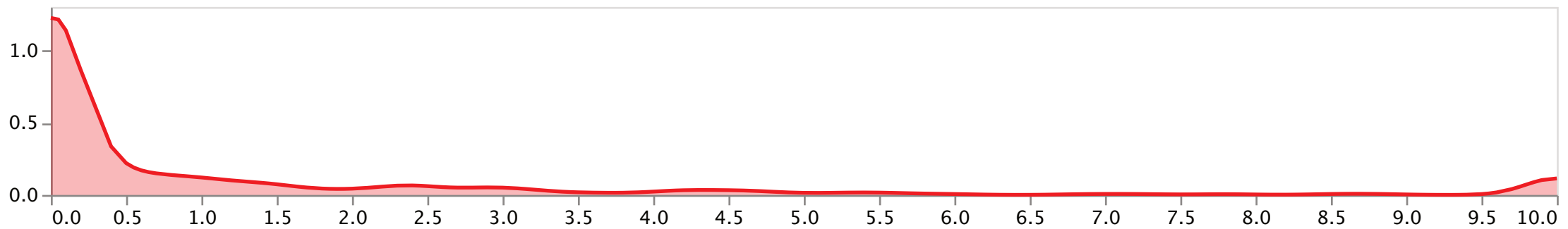

C.

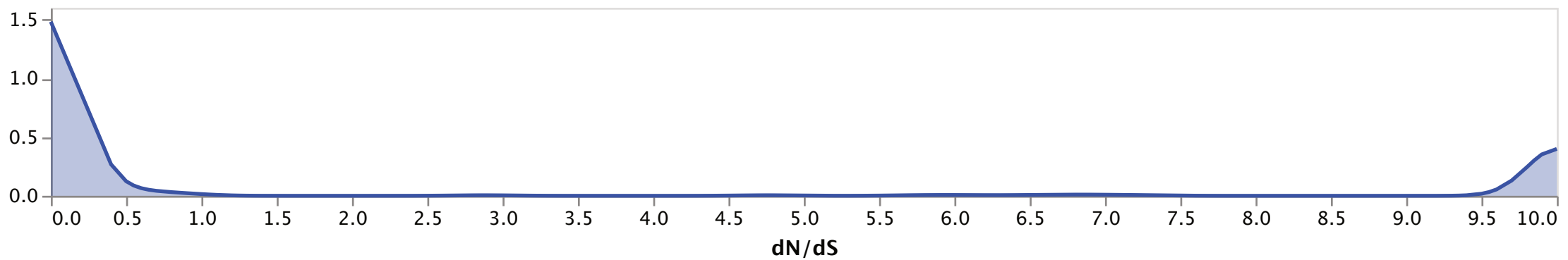

Supplement: S13 Fig — The black vertical bar indicates a neutral ratio. A) estimates from NP-like elements with reading frame disruptions of bats from Murina and Myotis; B) estimates from filovirus VP35-like elements (extended ORFs) in genomes of bats (Murina and Myotis); C) estimates from the filovirus NP-like elements in spalacid rodents with an open reading frame and expression products. (PDF) [file ppat.1011864.s013.pdf]

*Murina aurata*  
PVJC01004914

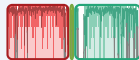

← EBOV VP35-like elements  
42842-43747

*Myotis lucifugus*  
NW\_005871048

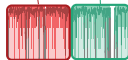

TRIM36

50 kbp

12239113-1344684

KCNN2

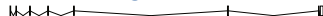

Supplement: S14 Fig — Colored boxes are local colinear blocks (aligned regions that lack internal rearrangements). Internal nucleotide similarity plots are shown inside the boxes (higher peaks are more similar). The graphs are positioned at the VP35-like insertions (location in each Accession is shown below the insert box) for comparison. Gene tracks are presented for the reference genome of Myotis lucifugus. Scale bar is 50 kbp. (PDF) [file ppat.1011864.s014.pdf]

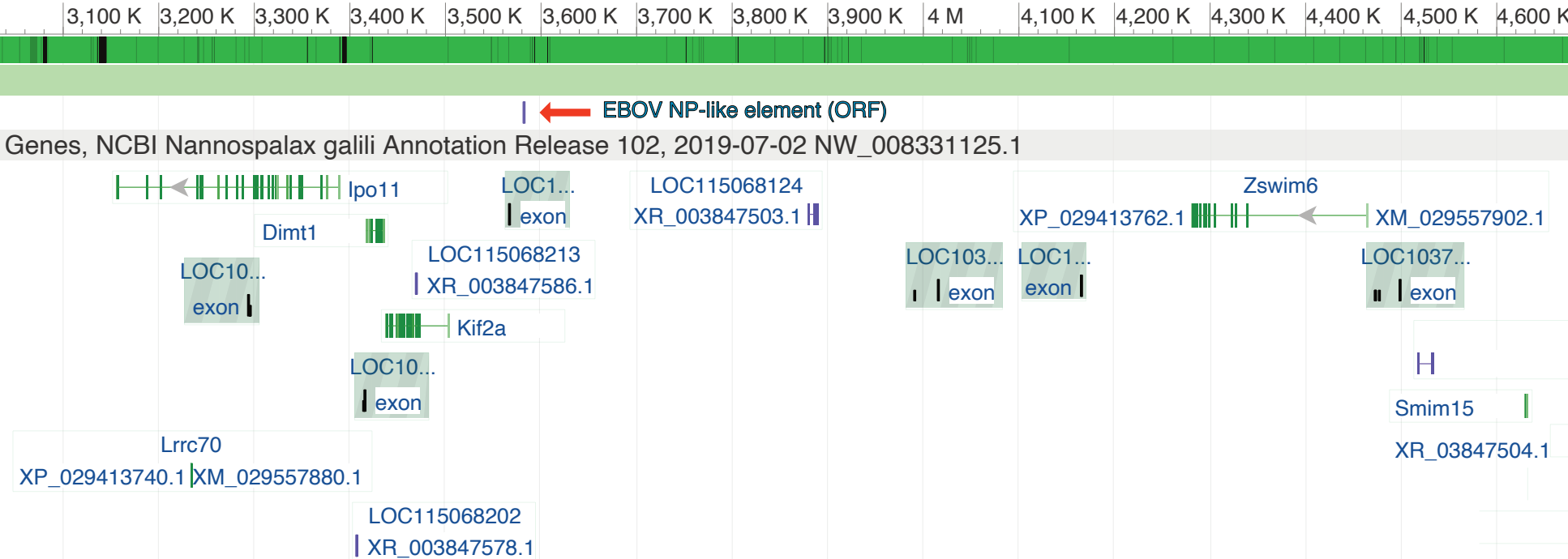

Supplement: S15 Fig — Gene tracks are presented below in green. Scale bar is above the green bar. (PDF) [file ppat.1011864.s015.pdf]

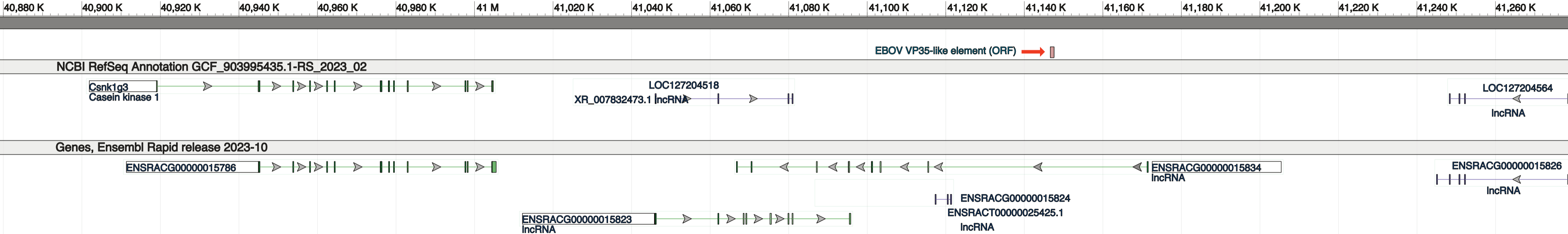

Supplement: S16 Fig — Gene tracks from NCBI Refseq and Ensembl are presented below in green and gray. Scale bar is above the dark gray bar. (PDF) [file ppat.1011864.s016.pdf]

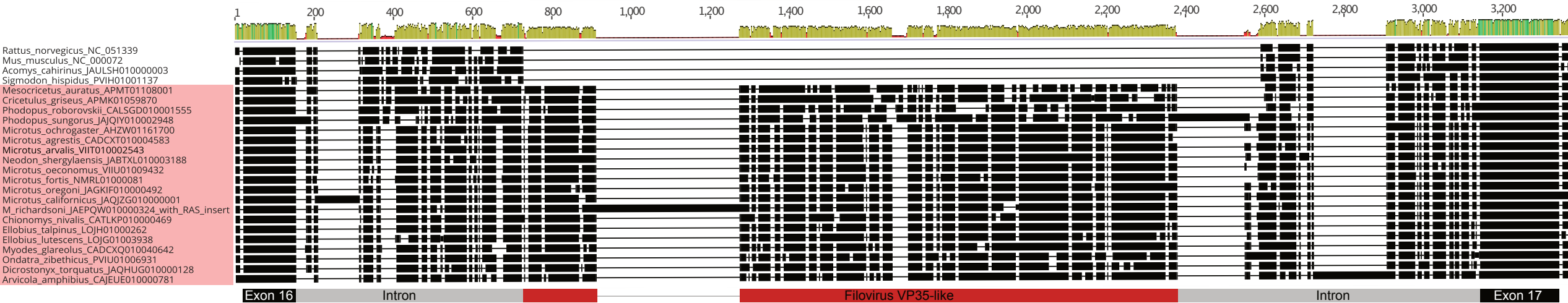

Supplement: S17 Fig — Dark shaded bars indicate exonic regions and gray shaded bars indicate the 3’ intron of Tax1bp1. The red bar below the alignment indicates region that has significant similarity to VP35 protein sequences of pathogenic filoviruses. Vertical bars above the alignment indicate sequence similarity (including differences in sequences that lack the insert). Genomic regions of four muroid rodents that lack the insert are shown for comparison. (PDF) [file ppat.1011864.s017.pdf]

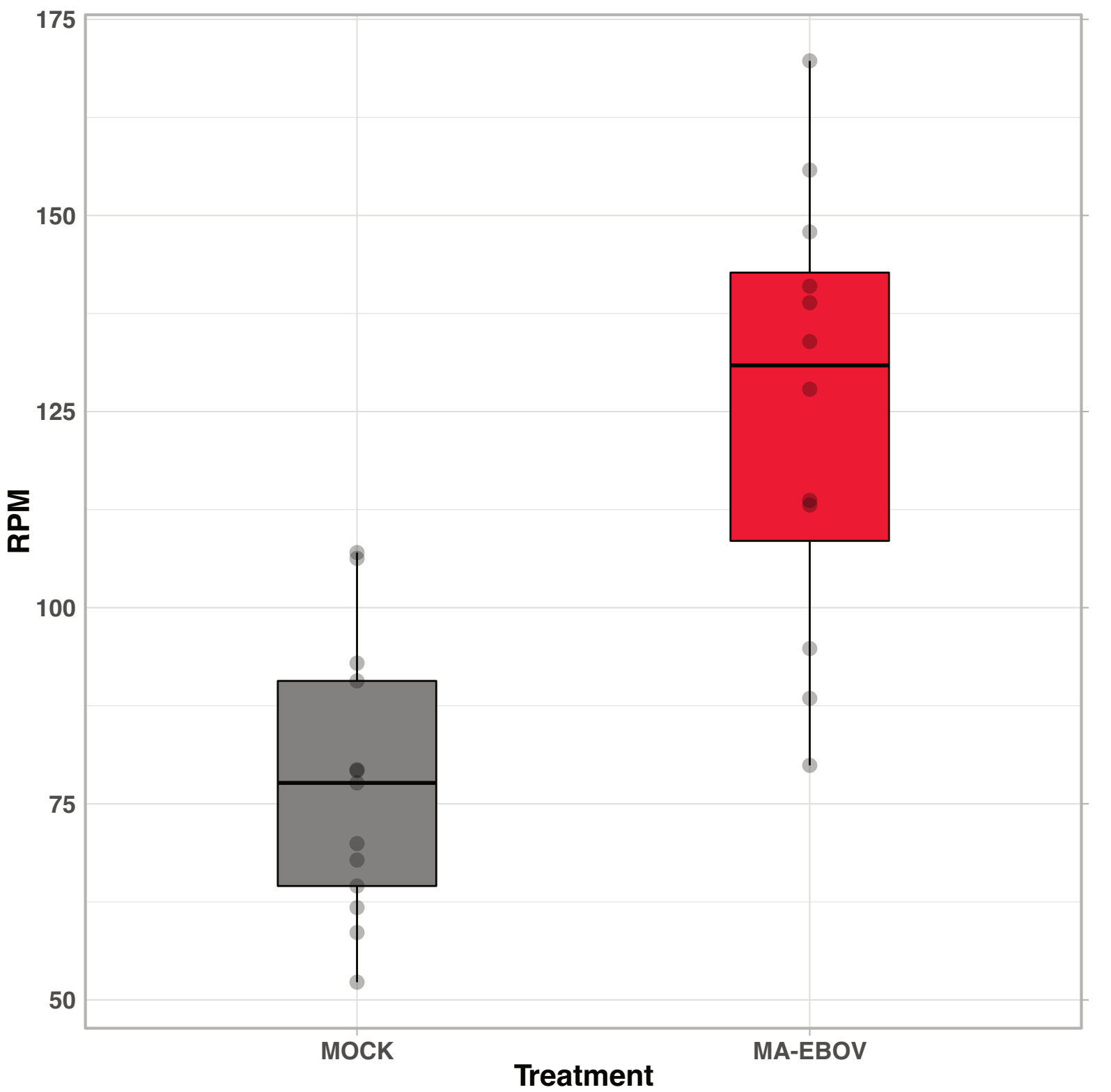

Supplement: S18 Fig — MA-EBOV are reads from livers of mice (5 days post infection with mouse adapted ebolavirus). Mock results are reads from livers of the same strains (5 days post mock infection). Reads are from SRA Project PRJNA540840 and the mapping results for each mouse are presented in S2 Table. The Y-axis is a normalized read map score, reads assigned per million reads (RPM). (PDF) [file ppat.1011864.s018.pdf]

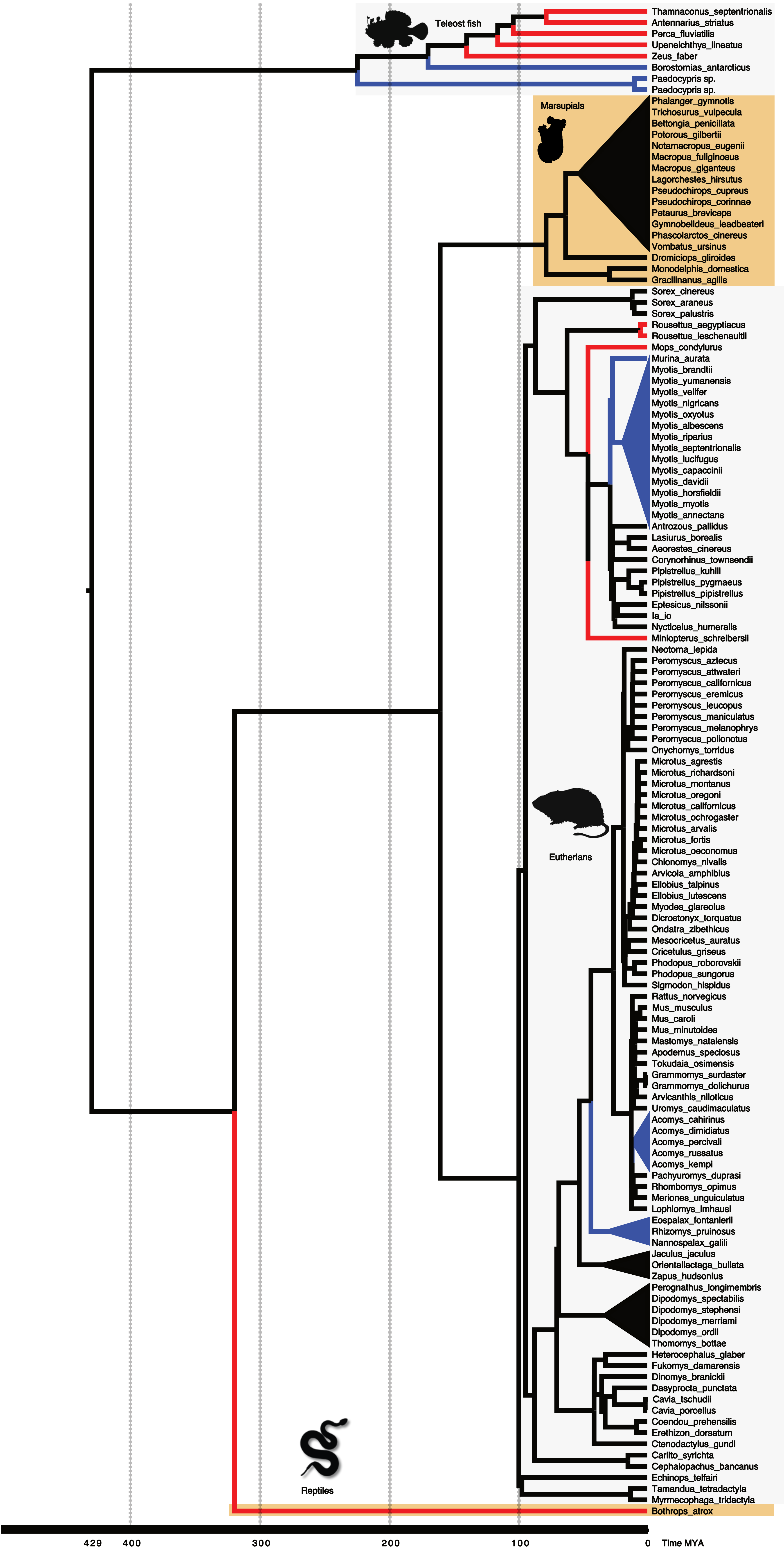

Supplement: S19 Fig — Boxes indicate major groups of vertebrates (teleost fish, reptiles, marsupials and eutherians). Vertical gray lines indicate 100 million-year intervals. Red branches lead to hosts with extant viral lineages, blue lines indicate vertebrates with paleoviral lineages that have extended open reading frames and black lines indicate vertebrates with paleoviral lineages that have only disrupted open reading frames. (PDF) [file ppat.1011864.s019.pdf]
