# Supplementary material for: Genomic transfers help to decipher the ancient evolution of filoviruses and interactions with vertebrate hosts
Source: PLoS Pathog. 2024 Sep 3;20(9):e1011864. doi: 10.1371/journal.ppat.1011864 (PMC11398700; doi:10.1371/journal.ppat.1011864)
Supplement: S1 Table — (PDF) [file ppat.1011864.s021.pdf]

	Taxon	Chi2 Composition test	p-value	
	1 NC_076535_Tapajosvirus_TAPV	passed	94.21%	
	2 Bundibugyo_ebolavirus	passed	66.35%	
	3 NC_016144_Lloviucuevavirus	passed	94.15%	
	4 NC_014372TaiForestebolavirus	passed	50.62%	
	5 NC_004161Restonebolavirus	passed	41.00%	
	6 NC_039345Bombaliebolavirus	passed	86.82%	
	7 NC_002549Zaireebolavirus	passed	69.51%	
	8 NC_006432Sudanebolavirus	passed	88.06%	
	9 NC_055510Mengladianlovirus	failed	0.00%	
	10 NC_001608Marburgmarburgvirus	failed	1.26%	
	11 NC_024781MarburgmarburgvirusRavn	passed	13.43%	
	12 NC_055175Wenlingfrogfishfilovirus_XILV	failed	0.37%	
	13 NC_076735Oberlandvirus	failed	0.12%	
	14 NC_055176Wenlingthamnaconusseptentrionalis	failed	0.01%	
	15 NC_076734_Fiwi_virus	failed	0.00%	
	16 NC_076916_Kander_virus	failed	0.00%	
	** TOTAL	7 sequences failed composition	chi2 test (p-value<5%; df=19)	
