# Supplementary material for: Genomic transfers help to decipher the ancient evolution of filoviruses and interactions with vertebrate hosts
Source: PLoS Pathog. 2024 Sep 3;20(9):e1011864. doi: 10.1371/journal.ppat.1011864 (PMC11398700; doi:10.1371/journal.ppat.1011864)
Supplement: S2 Table — MA_EBOV are reads from livers of mice (5 days post infection with mouse adapted ebolavirus). Mock results are reads from livers of the same strains (5 days post mock infection). Reads are from SRA Project PRJNA540840. RPM is a normalized read map score, reads assigned per million reads (RPM). (PDF) [file ppat.1011864.s022.pdf]

SRA Run	Reads	Reads mapped	Infection	strain	RPM
**SRR9003809**	50,190,359	7,423	MA_EBOV	CC041	147.89692976693
**SRR9003810**	91,867,558	12,952	MA_EBOV	CC041	140.985569682825
**SRR9003811**	42,809,576	6,669	MA_EBOV	CC041	155.782902404826
**SRR9003847**	53,127,601	4,245	MA_EBOV	CC055	79.9019703524727
**SRR9003848**	47,882,608	5,443	MA_EBOV	CC055	113.67384165875
**SRR9003849**	44,043,826	4,981	MA_EBOV	CC055	113.091900780827
**SRR9003634**	36,449,655	3,455	MA_EBOV	CC042	94.7882771455587
**SRR9003635**	41,613,949	5,779	MA_EBOV	CC042	138.871703812585
**SRR9003636**	40,384,045	5,163	MA_EBOV	CC042	127.847519979735
**SRR9003670**	48,133,169	4,257	MA_EBOV	CC043	88.4421302075498
**SRR9003671**	49,671,167	6,652	MA_EBOV	CC043	133.920751247902
**SRR9003672**	47,287,563	8,025	MA_EBOV	CC043	169.706355982016
**SRR9003818**	46,919,565	3,723	mock	CC041	79.3485617353869
**SRR9003819**	48,712,590	3,143	mock	CC041	64.5213075305583
**SRR9003820**	44,458,762	3,109	mock	CC041	69.9299724090383
**SRR9003823**	44,684,949	3,031	mock	CC041	67.8304455489028
**SRR9003824**	39,731,958	2,454	mock	CC041	61.7638828672878
**SRR9003856**	48,143,732	4,474	mock	CC055	92.9300620068257
**SRR9003857**	47,757,596	3,709	mock	CC055	77.6630381479001
**SRR9003616**	38,580,385	4,129	mock	CC042	107.023296942216
**SRR9003617**	46,544,832	4,220	mock	CC042	90.6652751480551
**SRR9003618**	39,403,529	4,188	mock	CC042	106.284896461939
**SRR9003652**	38,123,229	3,020	mock	CC043	79.2167945690015
**SRR9003653**	39,731,898	2,328	mock	CC043	58.5927
**SRR9003654**	46,381,898	2,424	mock	CC043	52.26176815
