# Supplementary material for: Genomic transfers help to decipher the ancient evolution of filoviruses and interactions with vertebrate hosts
Source: PLoS Pathog. 2024 Sep 3;20(9):e1011864. doi: 10.1371/journal.ppat.1011864 (PMC11398700; doi:10.1371/journal.ppat.1011864)
Supplement: S1 Data — The distance matrix was used for S12 Fig. (PDF) [file ppat.1011864.s023.pdf]

	EBOV	Myotis myotis	TAPV	Nannospax	Paedocypris	Peromyscus	MARV	MLAV	Acomys russatus	Phalanger	BOMV	Rhizomys	LLOV	XILV	BDBV	SUDV	TAFV	RESTV
EBOV																		
Myotis_myotis	0.958																	
TAPV	1.040	0.881																
Nannospalax	0.857	0.642	0.916															
Paedocypris	1.427	1.168	1.236	1.217														
Peromyscus	0.408	0.943	0.812	0.701	1.061													
MARV	0.444	0.549	0.836	0.787	1.109	0.549												
MLAV	0.305	0.860	0.891	0.665	1.116	0.453	0.191											
Acomys russatus	0.446	1.029	0.969	0.692	1.094	0.453	0.585	0.517										
Phalanger	0.785	1.258	1.341	1.136	2.269	0.844	0.791	0.797	0.924									
BOMV	0.045	0.945	1.069	0.843	1.230	0.408	0.440	0.314	0.462	0.784								
Rhizomys	1.011	0.809	1.121	0.434	1.025	0.794	0.878	0.835	0.897	1.202	0.967							
LLOV	0.293	0.889	0.971	0.825	1.304	0.466	0.352	0.308	0.54	0.707	0.305	1.025						
XILV	1.617	1.458	1.587	1.228	0.615	1.184	1.432	1.486	1.406	2.028	1.271	1.227	1.369					
BDBV	0.059	0.914	1.034	0.839	1.484	0.406	0.466	0.312	0.428	0.773	0.065	1.015	0.281	1.342				
SUDV	0.056	0.972	1.105	0.854	1.515	0.405	0.456	0.307	0.458	0.793	0.056	1.009	0.301	1.529	0.059			
TAFV	0.063	0.926	1.030	0.833	1.488	0.402	0.463	0.300	0.448	0.788	0.065	1.022	0.280	1.263	0.03	0.058		
RESTV	0.077	0.893	1.028	0.850	1.211	0.392	0.449	0.288	0.443	0.777	0.067	1.008	0.288	1.538	0.063	0.054	0.064	
