# Supplementary material for: Genomic transfers help to decipher the ancient evolution of filoviruses and interactions with vertebrate hosts
Source: PLoS Pathog. 2024 Sep 3;20(9):e1011864. doi: 10.1371/journal.ppat.1011864 (PMC11398700; doi:10.1371/journal.ppat.1011864)
Supplement: S2 Data — (PDF) [file ppat.1011864.s024.pdf]

	EBOV	Myotis luc.	TAPV	Myotis capp	RAVN	MARV	MLAV	Acomys russatus	Trichosurus	Phodopus	Myo_ox	LLOV	Murina	BDBV	SUDV	TAFV	RESTV
EBOV																	
Myotis luc,	1.685																
TAPV	4.211	2.794															
Myotis capp	3.537	1.087	2.666														
RAVN	1.454	2.453	7.316	2.402													
MARV	2.946	1.509	3.277	1.814	1.034												
MLAV	1.648	1.869	4.306	3.050	1.372	2.407											
Acomys russatus	1.436	1.619	3.536	2.004	1.040	1.440	0.861										
Trichosurus	1.740	2.782	5.455	3.010	1.937	1.978	1.658	1.564									
Phodopus	15.733	12.629	12.513	14.415	12.135	11.807	13.476	12.46	14.039								
Myotis ox	2.372	1.446	2.978	2.116	1.769	1.485	4.979	4.692	2.936	12.825							
LLOV	0.517	3.580	3.766	3.329	1.676	1.934	1.723	1.096	1.145	12.694	4.290						
Murina	3.045	1.111	2.854	1.240	1.716	1.937	2.210	1.888	3.016	14.307	1.612	2.556					
BDBV	0.735	1.776	7.328	3.419	1.888	2.639	1.607	1.060	1.573	15.129	3.344	0.333	3.236				
SUDV	0.526	3.160	8.667	3.184	2.200	2.425	1.730	1.458	1.545	14.982	3.279	0.371	3.018	0.249			
TAFV	0.637	2.223	8.059	3.441	1.326	2.346	2.133	1.337	1.806	15.698	2.023	0.381	3.094	0.190	0.282		
RESTV	0.329	2.006	6.593	3.267	2.790	3.334	2.131	1.184	1.406	13.836	1.770	0.360	3.197	0.353	0.317	0.274	
