# Supplementary material for: Genomic transfers help to decipher the ancient evolution of filoviruses and interactions with vertebrate hosts
Source: PLoS Pathog. 2024 Sep 3;20(9):e1011864. doi: 10.1371/journal.ppat.1011864 (PMC11398700; doi:10.1371/journal.ppat.1011864)
Supplement: S12 Fig — Stop codons are depicted by an asterisk. Nine of the fifteen sequences are open reading frames. (PDF) [file ppat.1011864.s012.pdf]

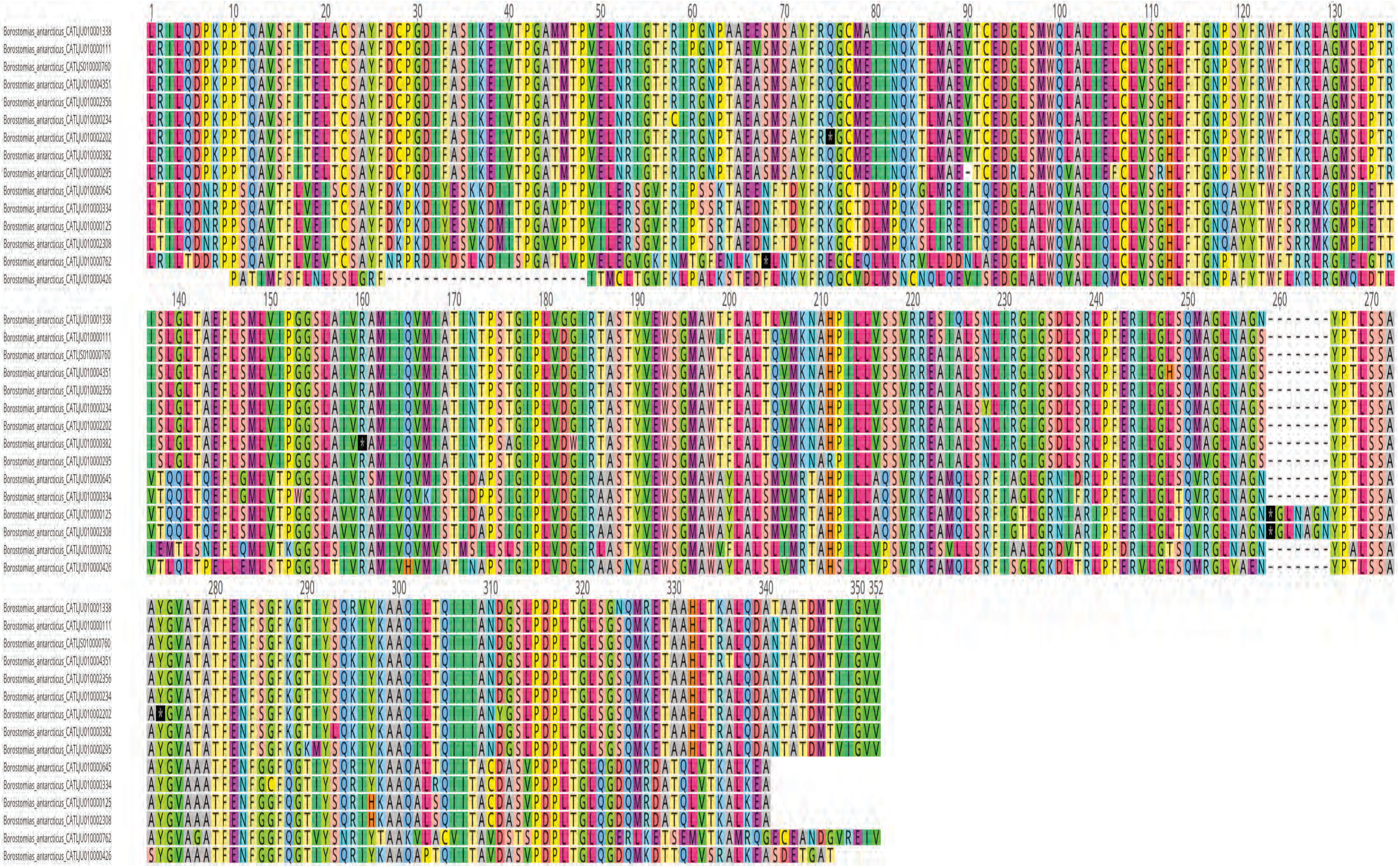
Borostomias_antarcticus_CATLJU010001338
Borostomias_antarcticus_CATLJU010000111
Borostomias_antarcticus_CATLJS010000760
Borostomias_antarcticus_CATLJU010004351
Borostomias_antarcticus_CATLJU010002356
Borostomias_antarcticus_CATLJU010000234
Borostomias_antarcticus_CATLJU010002202
Borostomias_antarcticus_CATLJU010000382
Borostomias_antarcticus_CATLJU010000295
Borostomias_antarcticus_CATLJU010000645
Borostomias_antarcticus_CATLJU010000334
Borostomias_antarcticus_CATLJU010000125
Borostomias_antarcticus_CATLJU010002308
Borostomias_antarcticus_CATLJU010000762
Borostomias_antarcticus_CATLJU010000426
1 10 20 30 40 50 60 70 80 90 100 110 120 130
140 150 160 170 180 190 200 210 220 230 240 250 260 270
280 290 300 310 320 330 340 350 352
